# Co-circulation of a Novel Dromedary Camel Parainfluenza Virus 3 and Middle East Respiratory Syndrome Coronavirus in a Dromedary Herd With Respiratory Tract Infections

**DOI:** 10.3389/fmicb.2021.739779

**Published:** 2021-12-07

**Authors:** Jade Lee Lee Teng, Ulrich Wernery, Hwei Huih Lee, Joshua Fung, Sunitha Joseph, Kenneth Sze Ming Li, Shyna Korah Elizabeth, Jordan Yik Hei Fong, Kwok-Hung Chan, Honglin Chen, Susanna Kar Pui Lau, Patrick Chiu Yat Woo

**Affiliations:** ^1^Department of Microbiology, Li Ka Shing Faculty of Medicine, The University of Hong Kong, Hong Kong, Hong Kong SAR, China; ^2^Central Veterinary Research Laboratory, Dubai, United Arab Emirates

**Keywords:** camel calves, metagenomics, Middle East respiratory syndrome coronavirus, novel species, dromedary camel parainfluenza virus 3, respiratory tract infections

## Abstract

Since the emergence of Middle East Respiratory Syndrome (MERS) in 2012, there have been a surge in the discovery and evolutionary studies of viruses in dromedaries. Here, we investigated a herd of nine dromedary calves from Umm Al Quwain, the United Arab Emirates that developed respiratory signs. Viral culture of the nasal swabs from the nine calves on Vero cells showed two different types of cytopathic effects (CPEs), suggesting the presence of two different viruses. Three samples showed typical CPEs of Middle East respiratory syndrome (MERS) coronavirus (MERS-CoV) in Vero cells, which was confirmed by partial RdRp gene sequencing. Complete genome sequencing of the three MERS-CoV strains showed that they belonged to clade B3, most closely related to another dromedary MERS-CoV isolate previously detected in Dubai. They also showed evidence of recombination between lineages B4 and B5 in ORF1ab. Another three samples showed non-typical CPEs of MERS-CoV with cell rounding, progressive degeneration, and detachment. Electron microscopy revealed spherical viral particles with peplomers and diameter of about 170nm. High-throughput sequencing and metagenomic analysis showed that the genome organization (3'-N-P-M-F-HN-L-5') was typical of paramyxovirus. They possessed typical genome features similar to other viruses of the genus *Respirovirus*, including a conserved motif ^323^FAPGNYALSYAM^336^ in the N protein, RNA editing sites 5'-^717^AAAAAAGGG^725^-3', and 5'-^1038^AGAAGAAAGAAAGG^1051^-3' (mRNA sense) in the P gene with multiple polypeptides coding capacity, a nuclear localization signal sequence ^245^KVGRMYSVEYCKQKIEK^261^ in the M protein, a conserved sialic acid binding motif ^252^NRKSCS^257^ in the HN protein, conserved lengths of the leader (55nt) and trailer (51nt) sequences, total coding percentages (92.6–93.4%), gene-start (AGGANNAAAG), gene-end (NANNANNAAAAA), and trinucleotide intergenic sequences (CTT, mRNA sense). Phylogenetic analysis of their complete genomes showed that they were most closely related to bovine parainfluenza virus 3 (PIV3) genotype C strains. In the phylogenetic tree constructed using the complete L protein, the branch length between dromedary camel PIV3 (DcPIV3) and the nearest node is 0.04, which is >0.03, the definition used for species demarcation in the family *Paramyxoviridae*. Therefore, we show that DcPIV3 is a novel species of the genus *Respirovirus* that co-circulated with MERS-CoV in a dromedary herd in the Middle East.

## Introduction

Camels are one of the most unique mammals on earth that have shown adaptation to desert life. There are three surviving Old World camel species, namely, *Camelus dromedarius* (dromedary or one-humped camel), which inhabits the Middle East, North, and Northeast Africa; *Camelus bactrianus* (Bactrian or two-humped camel) and *Camelus ferus* (the wild camel), both, inhabitants of Central Asia. Among the 20million camels on earth, 90% are dromedaries. Before the emergence of the Middle East Respiratory Syndrome (MERS) in 2012, viruses of at least eight families, including *Paramyxoviridae*, *Flaviviridae*, *Herpesviridae*, *Papillomaviridae*, *Picornaviridae*, *Poxviridae*, *Reoviridae*, and *Rhabdoviridae*, were known to infect dromedaries (Yousif et al., 2004; Wernery et al., 2008; Intisar et al., 2009; Khalafalla et al., 2010; Ure et al., 2011; Al-Ruwaili et al., 2012; Wernery et al., 2014). Subsequently, Middle East respiratory syndrome (MERS) coronavirus (MERS-CoV) was confirmed to be the causative agent of MERS (Lau et al., 2016a, 2017; El-Kafrawy et al., 2019; Al-Shomrani et al., 2020). In the last few years, we have discovered a number of novel viruses in dromedaries, including another coronavirus, named dromedary camel coronavirus UAE-HKU23, two novel genotypes of hepatitis E virus, a novel genus of enterovirus, a novel astrovirus, two novel bocaparvoviruses, and novel picobirnaviruses and circoviruses in dromedaries (Woo et al., 2014, 2015a,b, 2016, 2017; Sridhar et al., 2017). In addition, we have also described the first isolation of Newcastle disease virus and West Nile virus from dromedaries (Joseph et al., 2016; Teng et al., 2019).

In 2015, a herd of dromedaries consisting of nine camel calves from Umm Al Quwain, the United Arab Emirates, developed respiratory signs. Viral culture of the respiratory samples showed two different types of cytopathic effects (CPEs), suggesting the presence of two different viruses. To confirm the identities of these viruses, complete genome sequencing, phylogenetic, and comparative genome analyses were conducted.

## Materials and Methods

### Sample Collection and Viral Culture

In May 2015, nine camel calves (4–8months old) of the same herd developed respiratory signs with clear nasal discharge and fever. Nasal swab samples were collected and sent to the Central Veterinary Research Laboratory in Dubai, the United Arab Emirates for investigations. Nasal swab samples from the nine camel calves were inoculated into Vero cells, respectively, for isolation of MERS-CoV as previously described (Wernery and Zachariah, 1999; Wernery et al., 2015). Briefly, the samples were diluted 10-fold with viral transport medium and filtered. Two hundred microliters of the filtrate were inoculated into 200μl of minimum essential medium (Gibco, United States). Four hundred microliters of the mixture were added to 24-well tissue culture plates with Vero cells by adsorption inoculation. After 1h of adsorption, excess inoculum was discarded, the wells were washed twice with phosphate-buffered saline, and the medium was replaced with 1ml of minimum essential medium (Gibco, United States) supplemented with 1% fetal bovine serum (Gibco, United States). Culture was incubated at 37°C with 5% CO_2_ and inspected daily for CPEs for 7days by inverted microscopy. Two different types of CPEs were observed. All cultures with CPEs were screened for the presence of MERS-CoV using RT-PCR assay as described below.

### RNA Extraction

Viral RNA was extracted from the nine nasal swab samples and the corresponding culture samples using EZ1 Virus Mini Kit v2.0 (Qiagen, Hilden, Germany). RNA was eluted in 60μl of AVE buffer (Qiagen, Hilden, Germany) and was used as template for RT-PCR.

### RT-PCR for MERS-CoV

Screening of MERS-CoV was performed by amplifying a 440-bp fragment of the RdRp gene of CoVs using conserved primers (5'-GGTTGGGACTATCCTAAGTGTGA-3' and 5'-ACCATCATCNGANARDATCATNA-3') as described previously (Lau et al., 2016a).

### Complete Genome Sequencing of MERS-CoVs Detected From the Camel Calves

Three MERS-CoVs (D1189.1, D1189.5, and D1189.6), which showed typical CPE in Vero cells, isolated from three nasal swab samples from three dromedary calves were included in this study. One complete genome of MERS-CoV strain D1189.1 was sequenced in our previous study (Lau et al., 2016a). The complete genomes of the other two MERS-CoVs strains, D1189.5 and D1189.6, were sequenced in this study as previously described (Lau et al., 2016a). Briefly, the RNA extracted from the two MERS strains was converted to cDNA by a combined random-priming and oligo(dT) priming strategy. The cDNA was amplified by primers designed based on multiple sequence alignments of available MERS-CoV genome sequences using previously described strategies (Lau et al., 2016a). Primers used for PCR amplification and DNA sequencing were shown in Supplementary Table S1. Sequences were assembled and manually edited to produce the final sequences of the viral genomes using Geneious Prime 2020 (Kearse et al., 2012).

### Recombination Analysis

Bootscan analysis was performed to detect possible recombination by using the complete nucleotide alignment of the genome sequences of MERS-CoV and Simplot version 3.5.1, as previously described (Woo et al., 2006; Lau et al., 2011). The analysis was conducted using model F84, a sliding window of 1,500 nucleotides moving in 200 nucleotide steps with complete genome sequences D1189.1, D1189.5, and D1189.6, respectively, as the query. Possible recombination sites suggested by the bootscan analysis were confirmed through multiple sequence alignments.

### Sample Preparation for Illumina Sequencing

Three culture isolates (D1189.2, D1189.4, and D1189.8), which showed non-typical CPE in Vero cells, isolated from three nasal swab samples from three dromedary calves were negative for MERS-CoV using RT-PCR assay and subjected to further investigation by deep sequencing and microscopic analysis as described below. RNA was individually extracted from the three cultures, and the RNA samples were subjected to library preparation and Illumina sequencing, respectively, using NovaSeq 6000 (Pair-End sequencing of 151bp) at University of Hong Kong, Centre for Genomic Sciences (HKU, CGS), as described previously (Joseph et al., 2016).

### Sequence Analysis and *de novo* Assembly of Reads From Viruses of Interest

Illumina sequence raw reads were quality and adapter trimmed using Trimmomatic-0.4.3 with Nextera-PE FASTA sequences (Illumina, San Diego, CA, United States). Trimmed paired-end reads were analyzed as described previously (Joseph et al., 2016). The taxonomical content of the dataset was visualized by a phylogenetic tree computed using MEtaGenome ANalyzer (MEGAN) version 6.20.14, which assigned each sequence according to its taxonomical identity that are based on NCBI database (Huson et al., 2016). Once viruses of interest were found in the phylogenetic tree by MEGAN analysis, sequenced reads from the corresponding virus family, genus, or species were extracted. The extracted paired-end reads were *de novo* assembled into contigs using MIRA version 4.9.6 in accurate mode (Chevreux et al., 1999). The assembled contigs were subjected to further genome analysis by comparing with their corresponding closest relatives.

### Genome and Phylogenetic Analyses

The putative open reading frames (ORFs) and their deduced amino acid sequences of the assembled genomes were predicted using ORF Finder.^1^ The nucleotide sequences of the genomes and the deduced amino acid sequences of the ORFs were compared to those of other known viruses using ClustalOmega by multiple sequence alignment (Sievers and Higgins, 2014). MEGAX was used for the phylogenetic analyses of MERS-CoV (complete genome sequence) and dromedary camel parainfluenza virus 3 (DcPIV3; complete genome sequence, partial and complete amino acid sequence of L protein, and complete nucleotide sequences of L gene; Kumar et al., 2018). To minimize the potential loss of phylogenetic information in the trees constructed based on the amino acid sequences, both amino acid and nucleotide sequences were used for analyses. Maximum-likelihood method was used because it can apply a model of sequence evolution, representing a more accurate method than distance-based method for building a phylogeny using sequence data. The best substitution model for each alignment was predicted using the function “Find best DNA/Protein Model (Maximum-Likelihood)” implemented in MEGAX. Sequences of MERS-CoV were aligned using default parameter in MUSCLE. Phylogenetic tree of MERS-CoV was built with the model Tamura-Nei (TN93)+G+I where all sites were used for gaps and missing data. All the respective DcPIV3 sequences were aligned using MUSCLE with gap-opening penalty of five and gap extension penalty of one. Phylogenetic analyses of DcPIV3 were constructed using MEGAX with substitution model Jukes and Cantor (JC) with uniform rates for complete genome, Jones Taylor Thornton (JTT) with uniform rates for both partial and complete L proteins, and General Time Reversible (GTR)+G where all sites were used for gaps and missing data for complete L gene. Bootstrap analysis was performed for the assessment of confidence level of the observed clades in the inferred phylogenetic trees, in which 1,000 pseudoreplicates were used due to restrictions imposed by computational demand.

### Electron Microscopy

Dromedary camel parainfluenza virus 3 isolated from the sample D1189.8 was subjected to negative-contrast electron microscopy analysis as described previously (Lau et al., 2012; Lau et al., 2016b). Briefly, tissue culture cell extracts infected with DcPIV3 were centrifuged at 5,000×*g* at 4°C, after which the solution was fixed with GTA at a final concentration of 2.5% overnight. The sample was mounted into a carbon-formvar coated copper grid and stained with 3% urabyl acetate. The grid was then dried and irradiated with UV (1,250mW) for 15min with a Philips CM100 transmission electron microscope (Eindhoven, Netherlands).

### Nucleotide Sequence Accession Numbers

The nucleotide sequences of the two dromedary MERS-CoV genomes and the three DcPIV3 genomes sequenced in this study have been submitted to GenBank sequence database under accession numbers MW545527, MW545528, MW504257, MW504258, and MW504259. Raw data have been submitted to Sequence Read Archive (SRA) under accession numbers SRR13442189, SRR13442188, and SRR13442187.

## Results

### RT-PCR, Virus Culture, and Electron Microscopy

Nasal swab samples from nine camel calves of the same herd were collected and cultured on Vero cells for MERS-CoV screening. Three samples, D1189.1, D1189.5, and D1189.6, showed typical CPEs of MERS-CoV in Vero cells on day 4. RT-PCR targeting the 440-bp fragment of the RdRp gene confirmed the presence of MERS-CoV in these three nasal swab samples and their corresponding culture samples.

**Figure 1:**
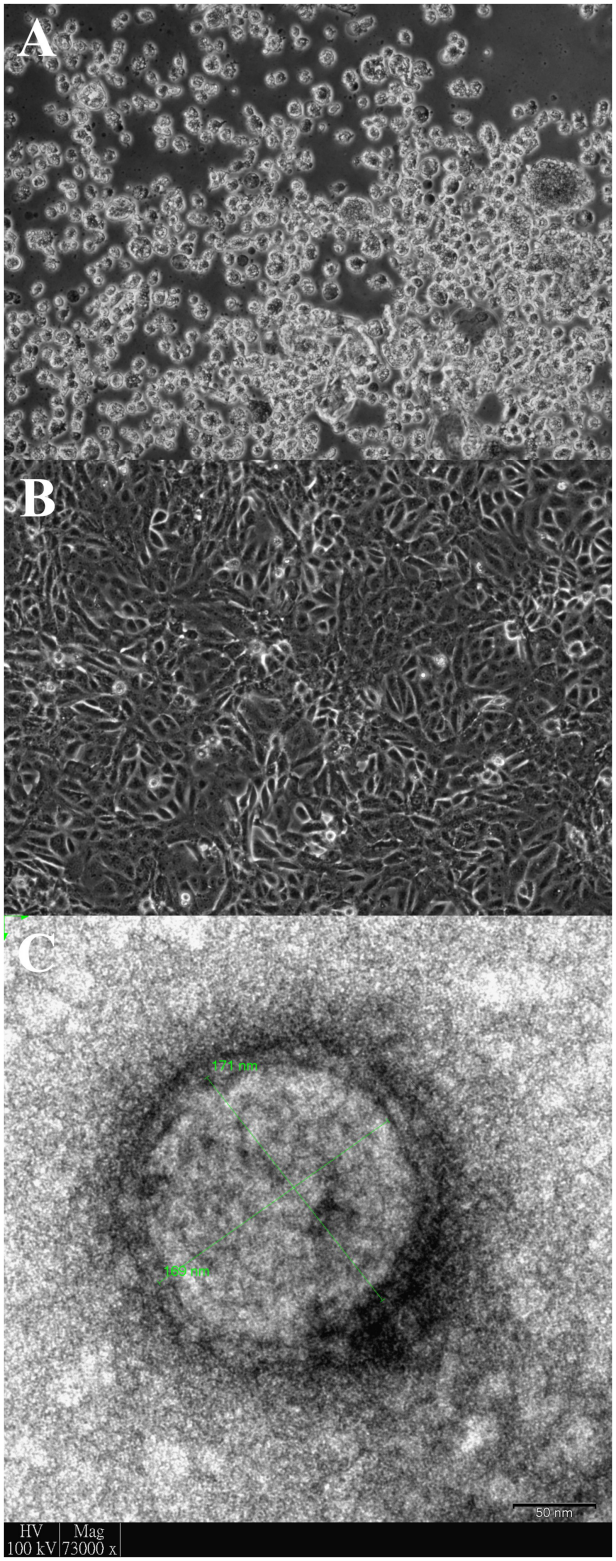
Vero cells infected with dromedary camel parainfluenza virus 3 (DcPIV3; D1189.8). Light micrograph showing cytopathic effects (CPEs) of D1189.8 culture isolate from the camel calf of a dromedary on **(A)** Vero cells; **(B)** Uninfected control of Vero cells. Magnification: ×100 and **(C)** DcPIV3 (D1189.8) viral particle stained with phosphotungstic acid under transmission electron microscope. The size of the displayed viral particle is about 170nm. Scale bar represents 50nm.

Another three cultures inoculated with samples D1189.2, D1189.4, and D1189.8 showed non-typical CPEs of MERS-CoV on day 4 with cell rounding, progressive degeneration, and detachment (Figures 1A,B). Electron microscopy of one of the three culture samples D1189.8 showed spherical viral particles with peplomers and diameter of about 170nm (Figure 1C). The CPE, morphology, and size of the virus were inconsistent with those of MERS-CoV. These three unknown culture isolates were subjected to high-throughput sequencing to confirm the presence of other viruses. The remaining three samples did not show any CPE in Vero cells. Therefore, further investigation on these samples was not proceded.

### Complete Genome Sequencing and Phylogenetic Analysis of MERS-CoV

**Figure 2:**
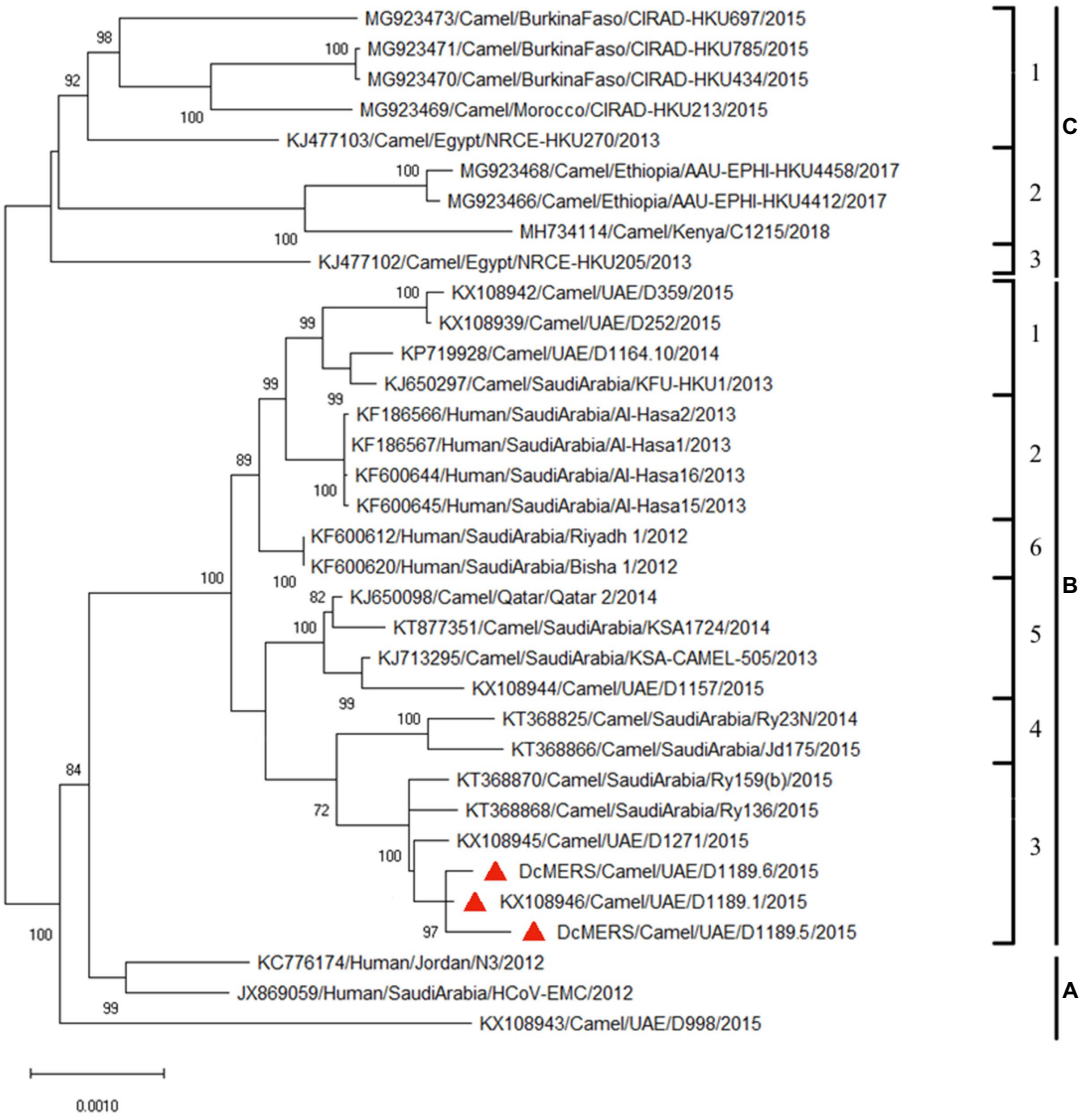
Maximum-likelihood phylogeny based on the complete genome sequences of the three isolated dromedary Middle East respiratory syndrome coronavirus (MERS-CoV) strains (D1189.1, D1189.5, and D1189.6) and those of other closely related MERS-CoV strains. Phylogenetic tree was generated using the TN93 model and a discrete Gamma distribution with 1,000 bootstrap pseudoreplicates. Thirty thousand three hundred thirty nucleotide positions of the complete genome were included in the analyses. The tree with the highest log likelihood (−48750.09) is shown. Bootstrap values are shown next to the branches. The scale bar indicates the number of nucleotide substitutions per site. The three MERS-CoV strains sequenced and analyzed in the present study are shown in red triangular. All the accession numbers are given as cited in GenBank.

Analysis of the complete genomes of the three isolated dromedary MERS-CoV strains (D1189.1, D1189.5, and D1189.6) showed that these sequences were 30,103 bases in length with G+C content of 41.1%. The size, G+C content, and genome structure of the three isolated dromedary MERS-CoV strains are similar to other dromedary MERS-CoVs. The genome sequence analysis showed that the three MERS-CoV isolates were closely related among each other, sharing 99.88–99.95% nucleotide identities. Phylogenetic analysis of the complete genomes of the three MERS-CoV isolates showed that they belonged to clade B3, being most closely related to another dromedary isolate, D1271 (GenBank accession number KX108945), which was previously detected in Dubai and they shared 99.84–99.93% nucleotide identities (Figure 2). Comparison of deduced amino acid sequences of proteins among the three MERS-CoV isolates showed only 8–17 amino acid substitutions along the whole-genome sequences, most occurring in the membrane protein (M; Table 1), while 5–21 substitutions compared to other clade B3 strains (Figure 2).

**Table 1:** Comparison of amino acid substitutions among the three DcMERS-CoV isolates in this study.

Protein	Position (aa)	Strain		
		D1189.1	D1189.5	D1189.6
ORF1a	1,578	V	L	V
	1,666	M	M	I
	2,123	V	A	A
	2,241	S	S	P
	2,702	Q	H	Q
ORF1b	1,573	Q	Q	H
	1,934	C	Y	C
Spike (S)	1,188	G	G	S
	1,251	F	S	S
ORF4a	22	C	C	F
Membrane (M)	67	S	N	S
	77	Q	H	Q
	84	A	N	A
	85	A	G	A
	86	V	A	V
	127	T	N	T
	129	V	L	V
	136	S	F	S

### Recombinant Analysis

**Figure 3:**
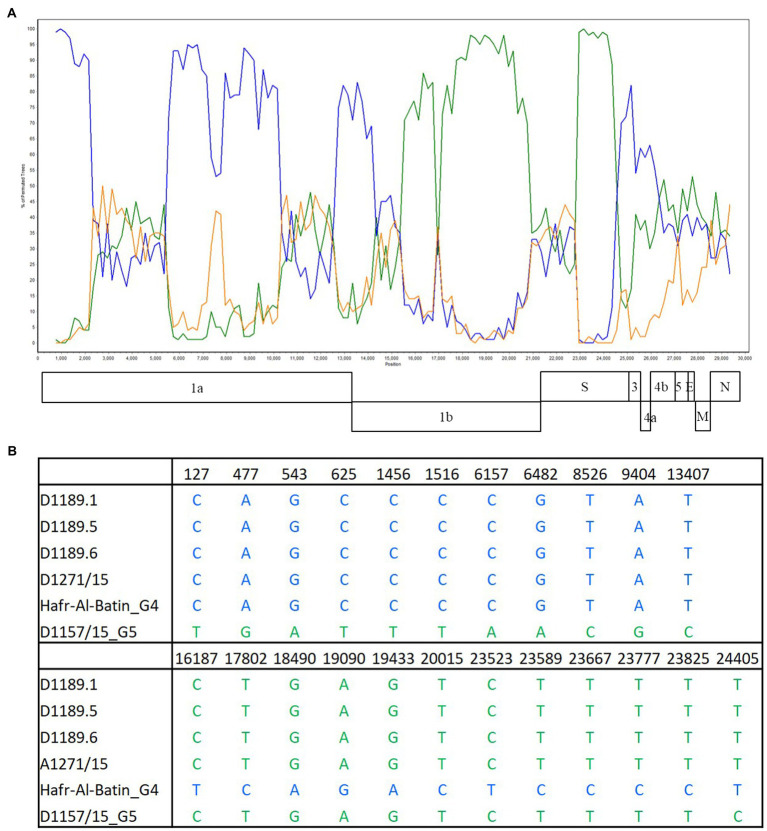
Detection of potential recombination by **(A)** bootscan analysis and **(B)** multiple sequence alignment. Bootscan analysis was conducted with Simplot version 3.5.1 (Maximum-likelihood; F84 model; window size 1,500bp; and step 200bp) on a gapless nucleotide alignment. D1189.1, D1189.5, D1189.6, and D1271 were selected as the query sequences and compared with the genome sequences of a lineage 4 MERS-CoV strain Hafr-Al-Batin (blue, KF600628), a lineage 5 MERS-CoV strain D1157/15 (green, KX108944), and a lineage 1 MERS-CoV strain UAE/Abu Dhabi_UAE_9 (orange, KP209312).

Bootscan analysis showed high bootstrap frequencies (80–100%) for clustering between the three strains and lineage 4 MERS-CoV in their genomes (position 1–14,000); but for position 15,000–24,000, bootscan analysis showed high bootstrap frequencies (75–100%) for clustering between the three strains and lineage 5 MERS-CoV (Figure 3A). Additional multiple sequence alignment using the three strains, a lineage 4 MERS-CoV and a lineage 5 MERS-CoV indicated that upstream of position 13,407, the three strains possessed nucleotides identical to lineage 4 MERS-CoV; but from position 16,187–23,825, the three strains possessed nucleotides similar to lineage 5 MERS-CoV (Figure 3B).

### Metagenomics Analysis of the Three Unknown Culture Isolates

The three culture isolates (D1189.2, D1189.4, and D1189.8) showed non-typical CPEs of MERS-CoV on Vero cells were subject to high-throughput sequencing, respectively, generating 5,621,614–6,500,327 paired-end 151-bp reads. After trimming adapter sequences and filtering the rRNA sequences, bacterial, and host genomes, a total of 4,377,997–5,432,096 clean reads remained and were used for downstream BLASTx analysis. Among these clean reads, 82,373–94,948 reads matched to viruses. The largest portion of these viral sequences was assigned to the family *Paramyxoviridae* (*n*=54,570–94,941). *De novo* assembly of reads of *Paramyxoviridae* revealed a complete genome of parainfluenza virus 3 (PIV3) of the genus *Respirovirus* in all three samples, tentatively named dromedary camel parainfluenza virus 3 (DcPIV3), where DcPIV3-1189.2, DcPIV3-1189.4, and DcPIV3-1189.8 represented the parainfluenza virus 3 discovered in the sample D1189.2, D1189.4, and D1189.8, respectively. RT-PCR targeting 400-bp fragment of the F gene confirmed the presence of DcPIV3 in these three nasal swab samples and their corresponding culture samples.

### Genome and Phylogenetic Analysis of DcPIV3

**Figure 4:**
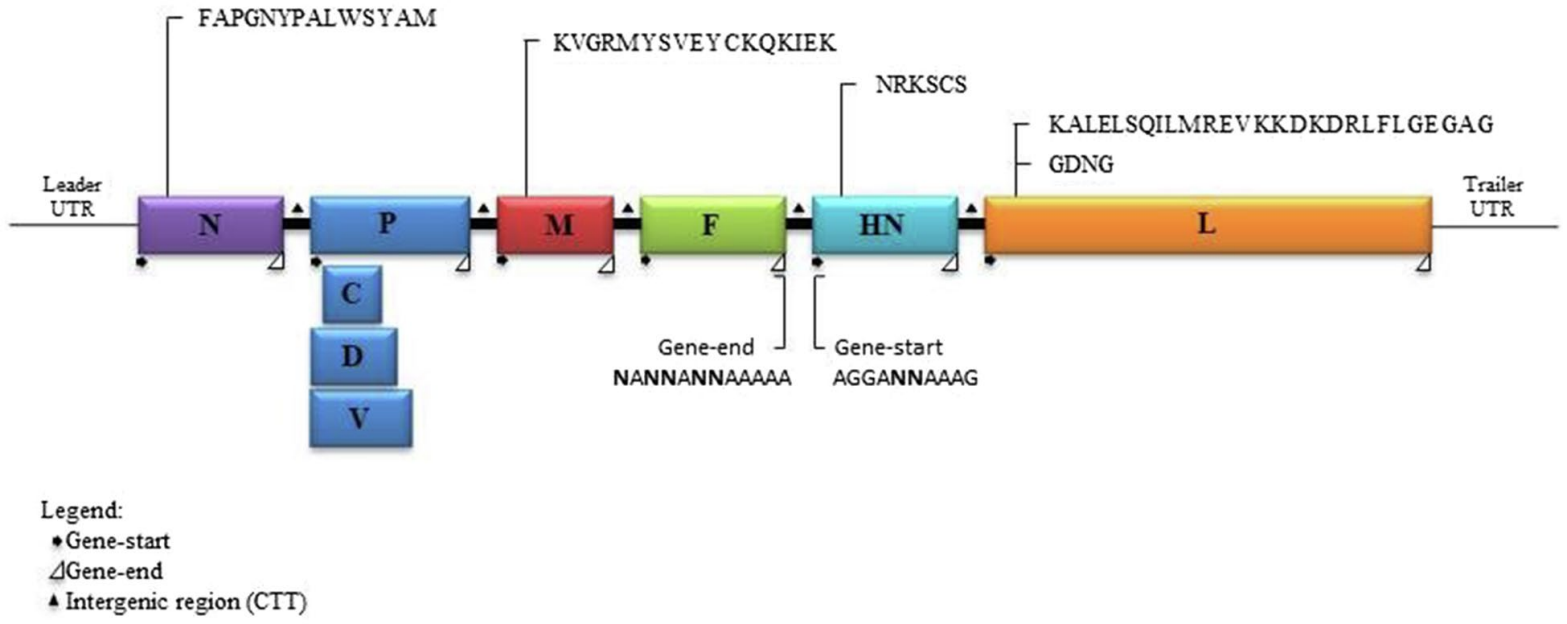
Schematic diagram of a DcPIV3 genome with the transcriptional gene-start, intergenic, and gene-end sequences. The gene sizes are shown as boxes that are drawn to approximate scale. The positions of the extragenic 3' leader, 5' terminal trailer, and gene junctions are shown as horizontal lines. The conserved gene-start and gene-end transcription regulatory sequences at the boundaries between genes are indicated.

Analysis of the complete genome of the three DcPIV3 strains showed that these sequences ranged from 15,474–15,498 bases in length, which conformed to the paramyxovirus rule of six, and had an overall G+C content of 34.6–34.8% (Table 2). They were highly similar and shared 98.8% nucleotide identities among each other (Table 3). Comparison of deduced amino acid sequences of proteins among the three DcPIV3 strains showed only 8–22 amino acid substitutions along the whole-genome sequences (Table 4). Overall, the genome organization of the three DcPIV3 strains was typical of paramyxovirus, with six genes 3′-N-P-M-F–HN-L-5′ encoding the nucleocapsid protein (N), phosphoprotein (P), matrix protein (M), fusion protein (F), hemagglutinin neuraminidase (HM), and large polymerase (L), respectively (Table 2; Figure 4). They possessed typical genome features similar to other viruses of the genus *Respirovirus*, including a conserved motif ^323^FAPGNYALSYAM^336^ in the N protein, RNA editing sites 5'–^717^AAAAAAGGG^725^–3' and 5'-^1038^AGAAGAAAGAAAGG^1051^-3' (mRNA sense) in the P gene with multiple polypeptides coding capacity, a nuclear localization signal sequence ^245^KVGRMYSVEYCKQKIEK^261^ in the M protein, a conserved sialic acid binding motif ^252^NRKSCS^257^ in the HN protein, conserved lengths of the leader (i.e., 55nt) and trailer (i.e., 51nt) sequences, total coding percentages (92.6–93.4%), gene-start (consensus: AGGANNAAAG), gene-end (consensus: NANNANNAAAAA), and trinucleotide intergenic sequences (i.e., CTT, mRNA sense; Table 2; Figure 4). Similar to P genes of respiroviruses, the P gene of DcPIV3 encodes for three overlapping polypeptides, including a non-structural C protein (203 aa utilizing +1 frame), a cysteine-rich V protein (158 aa + 1G), and a D protein (131 aa + 2G).

**Table 2:** Genomic features and coding potential of the three DcPIV3 strains isolated from dromedary nasal samples.

DcPIV3 strain			ORF features								
	Length (nt)	G+C content (%)	Protein	Location (nt)	Length (nt)	Length (aa)	Frame	mRNA insertion	Gene-start	IGR	Gene-end
*D1189.2*	15,474	34.8	Nucleoprotein (N)	112–1,659	1,548	516			AGGATTAAAG	CTT	GAGTAAGAAAAA
			Phosphoprotein (P)	1,785–3,587	1,803	601			AGGATTAAAG	CTT	TAATAATAAAAA
			C protein (C)	1,795–2,403	609	203	+1				
			D protein (D)	1,785–2,178	393	131		+2G			
			V protein (V)	1,785–2,259	474	158		+1G			
			Matrix (M)	3,748–4,803	1,056	352			AGGACAAAAG	CTT	AAAAATCAAAAA
			Fusion (F)	5,096–6,718	1,623	541			AGGATCAAAG	CTT	AAGTATAAAAAA
			Hemagglutinin neuraminidase (HN)	6,830–8,548	1,719	573			AGGAACAAAG	CTT	GAAAATAAAAAA
			Large (L)	8,670–15,371	6,702	2,234			AGGAGAAAAG	CTT	AAATAAGAAAAA
*D1189.4*	15,498	34.8	Nucleoprotein (N)	111–1,658	1,548	516			AGGATTAAAG	CTT	GAGTAAGAAAAA
			Phosphoprotein (P)	1,784–3,586	1,803	601			AGGATTAAAG	CTT	GATTAAGAAAAA
			C protein (C)	1,794–2,403	609	203	+1				
			D protein (D)	1,784–2,177	393	131		+2G			
			V protein (V)	1,784–2,258	474	158		+1G			
			Matrix (M)	3,747–4,802	1,056	352			AGGACAAAAG	CTT	AAAAATCAAAAA
			Fusion (F)	5,108–6,730	1,623	541			AGGATCAAAG	CTT	AAGTATAAAAAA
			Hemagglutinin neuraminidase (HN)	6,842–8,560	1,719	573			AGGAACAAAG	CTT	TAAAATAAAAAA
			Large (L)	8,694–15,395	6,702	2,234			AGGAGAAAAG	CTT	AAATAAGAAAAA
*D1189.8*	15,480	34.6	Nucleoprotein (N)	111–1,658	1,548	516			AGGAGAAAAG	CTT	GAGTAAGAAAAA
			Phosphoprotein (P)	1,784–3,586	1,803	601			AGGATTAAAG	CTT	TACTATGAAAAA
			C protein (C)	1,794–2,403	609	203	+1				
			D protein (D)	1,784–2,177	393	131		+2G			
			V protein (V)	1,784–2,258	474	158		+1G			
			Matrix (M)	3,747–4,802	1,056	352			AGGAGAAAAG	CTT	AAAAATCAAAAA
			Fusion (F)	5,102–6,724	1,623	541			AGGATCAAAG	CTT	AAATATAAAAAA
			Hemagglutinin neuraminidase (HN)	6,836–8,554	1,719	573			AGGAACAAAG	CTT	GAAAATAAAAAA
			Large (L)	8,676–15,377	6,702	2,234			AGGAGAAAAG	CTT	AAATAAAAAAAA

**Table 3:** Comparison of pairwise nucleotide identity between the three DcPIV3 strains isolated from dromedary nasal samples with other representative PIV3 strains.

PIV3 strain (GenBank accession no.)	Pairwise identity (%)		*D1189.2*	*D1189.4*	*D1189.8*
*Dromedary parainfluenza virus 3 (DcPIV3)*			
Camel D1189.2 (MW504257)		98.4	98.9
Camel D1189.4 (MW504258)	98.4		97.9
Camel D1189.8 (MW504259)	98.9	97.9	
*Bovine parainfluenza virus 3 (BPIV3)*			
*Genotype A*			
*Sub-genotype A1*			
Bovine TVMDL24 (KJ647288)	83.9	84.3	83.7
Bovine 910N (D84095)	84.3	84.3	83.7
*Sub-genotype A2*			
Bovine 15,626 (AF178654)	84.1	84.4	83.8
Bovine Shipping fever (AF178655)	83.7	84.4	83.9
*Sub-genotype A3*			
Bovine TVMDL60 (KJ647289)	83.9	84.2	83.6
Cattle NM09 (JQ063064)	83.9	84.1	83.6
*Genotype B*			
Bovine Q5592 (EU277658)	83.2	83.6	83.1
Bovine TVMDL15 (KJ647284)	83.4	83.7	83.2
Bovine TVMDL17 (KJ647286)	83.5	83.8	83.3
*Genotype C*			
Cattle 12Q061 (JX969001)	84.8	85.1	84.8
Cattle SD0835 (HQ530153)	85.2	85.4	84.9
Bovine TVMDL16 (KJ647285)	85.1	85.5	84.9
Bovine TVMDL20 (KJ647287)	85.2	85.5	85.0

**Table 4:** Comparison of amino acid substitutions among the three DcPIV3 isolates in this study.

Protein	Position (aa)	Strain	D1189.2	D1189.4	D1189.8
*P gene*	530	K	E	E
	756	R	G	G
	793	N	S	N
*M gene*	1,463	K	K	E
*F gene*	1,470	I	F	I
	1,476	I	V	I
	1,479	I	V	I
	1,693	T	K	K
	1,732	V	F	V
	1,734	D	V	D
	1,736	D	Y	D
	1,739	D	E	D
	1,788	F	F	S
	1,830	R	K	I
*HN gene*	2,198	V	A	A
	2,203	N	H	N
	2,260	K	K	R
	2,336	N	K	N
*L gene*	4,426	S	L	S
	4,441	Q	L	Q
	4,442	I	L	I
	4,446	V	E	V
	4,451	N	S	N
	4,461	N	I	N

**Figure 5:**
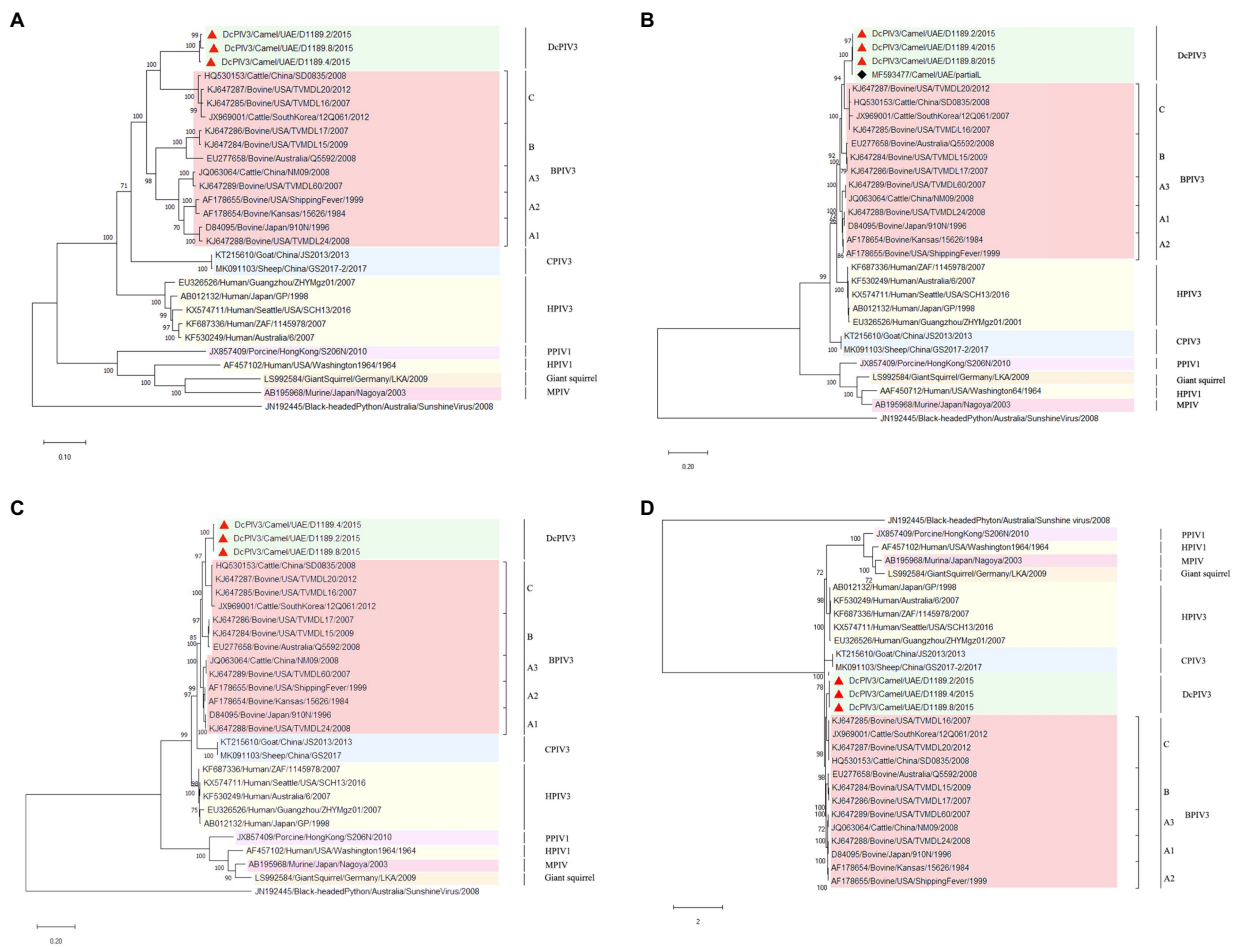
Phylogenetic analyses of **(A)** the complete genome, the amino acid sequences of **(B)** partial L protein and **(C)** complete L protein, and **(D)** the nucleotide sequences of the complete L gene of the three DcPIV3 strains discovered in the present study. Phylogenetic trees were generated using Jukes and Cantor (JC) model (complete genome), Jones Taylor Thornton (JTT) model (partial L and complete L protein), and General Time Reversible (GTR)+G model (complete L gene) with 1,000 bootstrap pseudoreplicates. Nineteen thousand and fifty-four nucleotide positions of the complete genome, 1,635 and 2,428 amino acid positions of the partial and complete L protein, and 5,861 nucleotide positions of the complete L gene were included, respectively, in the analyses. Trees with the highest log likelihood (−181887.15 for the complete genome, −17365.96 for the partial L protein, −27636.23 for the complete L protein, and −53701.29 for the complete L gene) are shown. Bootstrap values are shown next to the branches. The scale bar indicates the number of nucleotide or amino acid substitutions per site. The three DcPIV3 strains sequenced and analyzed in the present study are shown in red triangular and black spade for MF593477. All the accession numbers are given as cited in GenBank.

Phylogenetic analysis of the complete genome of the three sequenced DcPIV3 strains and representative viruses of the genus *Respirovirus* showed that they were most closely related to BPIV3 genotype C (BPIV3c) strains, being most closely related to BPIV3c strain TVMDL20 (GenBank accession number KJ647287), sharing 85.2–85.5% nucleotide identities (Table 3; Figure 5A). They shared 83.7–84.4, 83.1–83.8, and 84.8–85.5% nucleotide identities to other BIV3 strains of genotype A, B, and C, respectively (Table 3). Further sequence analysis of the L protein revealed that the three DcPIV3 strains shared 98.0% amino acid identities to the partial L protein (1,525 aa) of a PIV3 strain previously discovered from a MERS-CoV-positive dromedary camel in Abu Dhabi, the UAE (GenBank accession number MF593477; Figure 5B). Phylogenetic tree constructed based on the complete L protein showed that the branch length between the three DcPIV3 strains and the nearest node, BPIV3, was >0.03 (Figure 5C), and it showed similar topology as the trees constructed based on the whole-genome sequence (Figure 5A) and the nucleotide sequence of the complete L gene (Figure 5D). The pairwise amino acid identities of N, P, M, F, HN, and L of the three DcPIV3 strains and other virus strains of BPIV3 genotypes were shown in Table 5.

**Table 5:** Comparison of amino acid identities between the predicted open reading frames (ORFs) of three DcPIV3 strains and the corresponding proteins of other representative PIV3 strains.

Representative member		Pairwise amino acid identity (%)				
Genotype	Strain	Accession no.	D1189.2	D1189.4	D1189.8	
			N	P	M	F	HN	L	N	P	M	F	HN	L	N	P	M	F	HN	L
*Dromedary parainfluenza virus 3 (DcPIV3)*		
NA	D1189.2	MW504257							100.0	99.5	100.0	97.6	99.5	99.3	100.0	99.8	99.7	99.4	99.3	100.0
NA	D1189.4	MW504258	100.0	99.7	99.7	97.6	99.5	99.3							100.0	99.7	99.7	97.6	99.1	99.3
NA	D1189.8	MW504259	100.0	99.8	99.7	99.4	99.3	100.0	100.0	99.7	99.7	97.6	99.1	99.3						
*Bovine parainfluenza virus 3 (BPIV3)*																				
A	15,626	AF178654	89.7	67.0	95.4	85.7	81.5	91.0	89.7	67.3	95.4	84.4	81.3	90.3	89.7	67.1	95.2	85.7	81.5	91.0
B	Q5592	EU277658	89.3	68.7	94.3	82.4	80.4	91.5	89.3	69.2	94.3	82.0	80.2	90.9	89.3	68.8	94.0	82.4	80.4	91.5
C	12Q061	JX969001	91.5	70.5	95.7	85.2	83.0	90.9	91.5	71.0	95.7	84.4	83.0	90.2	91.5	70.7	95.4	85.2	82.7	90.9
C	SD0835	HQ530153	90.3	71.8	97.2	84.8	83.2	91.6	90.3	72.3	97.2	84.1	83.2	91.0	90.3	72.0	96.9	84.8	82.9	91.6
C	TVMDL16	KJ647285	91.1	71.3	97.2	85.8	83.2	92.0	91.1	71.8	97.2	84.8	83.2	91.3	91.1	71.5	96.9	85.8	82.9	92.0
C	TVMDL20	KJ647287	91.5	72.0	97.2	85.6	82.7	92.0	91.5	72.5	97.2	84.6	82.7	91.3	91.5	72.2	96.9	85.6	82.3	92.0

## Discussion

In this study, we showed that both MERS-CoV and DcPIV3 co-circulated in a dromedary herd in the Middle East. Since the emergence of MERS epidemic in human in 2012, detection of MERS-CoV and its antibodies has been reported in dromedaries in various countries in the Middle East and North Africa (Lau et al., 2016a). We have also detected MERS-CoV neutralizing antibodies in Bactrian and hybrid camels from Dubai (Lau et al., 2020), suggesting that camel is probably the reservoir for MERS-CoV. In this study, the herd was from a dromedary farm in Umm Al Quwain, the UAE. When the dromedaries in the herd developed respiratory signs that were not specific for a particular respiratory infection, nasal swabs were collected from them for viral culture, of which one form of CPE developed in samples from three dromedaries and another kind of CPE was observed in specimens of another three dromedaries. In the first three samples (D1189.1, D1189.5, and D1189.6), MERS-CoV was detected by RT-PCR using virus-specific primers, consistent with the typical CPE for MERS-CoV on Vero cells. Complete genome sequencing and phylogenetic analysis revealed that D1189.1, D1189.5, and D1189.6 were clustered (Figure 2), indicating that the virus had probably been transmitted from one camel to another within the herd. They belonged to clade B3 and were most closely related to another dromedary isolate D1271 previously detected in Dubai (Figure 2). Similar to D1271, bootscan analysis and multiple alignment revealed evidence of recombination for the three B3 strains, with the potential recombination site detected in ORF1ab (Figure 3). Although several studies have reported recombination among MERS-CoVs from different countries (Huang et al., 2016; Sabir et al., 2016), we could not exclude the possibility that the apparent recombination events may have been resulted from individual nucleotide mutations. Such recombination analyses are particularly complicated in RNA viruses, including MERS-CoV, which are known to have high mutation rate. The high frequency of mutations will increase the likelihood of convergent mutations, causing sequence similarities in divergent virus strains that can be misinterpreted as recombination events. Furthermore, most recombination analysis tools may not be able to distinguish between recombined and rapidly evolving sequences. Therefore, one should be cautious when determining whether phylogenetic discordant regions are attributable to recombination or to convergent mutations. Overall, comparative genome analysis showed that the amino acid of the three MERS-CoV isolates differed by 8–17 amino acids with the highest amino acid substitutions at M protein (Table 1). Notably, in two of the three MERS-CoV strains (D1189.1 and D1189.6), their M protein sequences were identical, but they differed from the third one (D1189.5) by eight amino acids (Table 1). However, for the other parts of the genome (ORF1a, ORF1b, ORF4a, and S), which showed variations among the three strains, there were seven amino acid differences between D1189.1 and D1189.6, five amino acid differences between D1189.1 and D1189.5, and eight amino acid differences between D1189.5 and D1189.6 (Table 1; Figure 2).

In the other three samples that showed CPE atypical for MERS-CoV, DcPIV3 was detected. Antibodies against PIV3 have been detected in dromedaries for a few decades (Van der Maaten, 1969; Nawal et al., 2003; Shaker et al., 2003). In 2009, a PIV3 was first described in the respiratory samples of dromedaries (Intisar et al., 2010). In that study, two lung specimens from dromedaries in slaughterhouses from Sudan with pneumonia outbreak were found to be RT-PCR positive for PIV3, although no sequencing results were described. Virologists have speculated that this PIV3 detected in dromedaries could be BPIV3 (Van der Maaten, 1969; Nawal et al., 2003; Shaker et al., 2003). In 2017, PIV3 sequences were found in the nasopharyngeal swabs of healthy dromedaries by metagenomic sequencing (Li et al., 2017). However, only one partial L sequence from this study was uploaded in GenBank. In the present study, for the three samples (D1189.2, D1189.4, and D1189.8) that showed CPE on Vero cells but were RT-PCR negative for MERS-CoV, the viral isolates were subjected to next-generation sequencing, using a strategy we previously employed for the detection of West Nile virus in a dromedary (Joseph et al., 2016). Overall, comparative genome analysis showed that the concatenated amino acids of the three DcPIV3 isolates differed by 8–22 amino acids (Table 4). They possessed typical genome features similar to other viruses of the genus *Respirovirus*, including a conserved motif in the N protein, RNA editing sites in the P gene, a nuclear localization signal sequence in the M protein, a conserved sialic acid binding motif in the HN protein, conserved lengths of the leader and trailer sequences, total coding percentages, gene-start and gene-end, and trinucleotide intergenic sequences (Table 2, Figure 4). Phylogenetic analysis revealed that the three strains were clustered (Figure 5), indicating that they were also a result of inter-camel transmission within the herd. Phylogenetic trees constructed using complete genome or L protein showed that although DcPIV3 is most closely related to the other three genotypes of BPIV3, it forms a cluster distinct from BPIV3 (Figure 5).

Complete genome sequencing and phylogenetic and comparative genome analysis showed that DcPIV3 is a novel species of the genus *Respirovirus*. According to the ICTV definition, in the genus *Respirovirus* under the subfamily *Orthoparamyxoviriniae* of the *Paramyxoviridae* family, there are six species, namely, BPIV3, human parainfluenza virus 1 and 3 (HPIV1 and HPIV3), porcine parainfluenza virus 1 (PPIV1), caprine parainfluenza virus 3 (CPIV3), and Sendai virus (SeV; Rima et al., 2019). For the BPIV3 species, the members were further sub-classified into three genotypes; most of them were from cattle, although there is no concrete definition on the criteria for genotype demarcation. In the present study, although our results showed that DcPIV3 is most closely related to BPIV3c strains, DcPIV3 constitutes a new species in the genus *Respirovirus* because in the phylogenetic tree constructed using the complete L protein, the branch length between DcPIV3 and the nearest node is 0.04, which is more than 0.03, the definition used for species demarcation in the family *Paramyxoviridae*.

## Conclusion

Collectively, our results showed that both MERS-CoV and DcPIV3 co-circulated in a dromedary herd in the Middle East and DcPIV3 is a novel species of the genus *Respirovirus*. The present study is the first that demonstrated isolation of a novel respirovirus in sick dromedaries, further expanding the host range for respiroviruses. Future studies are warranted to improve our understanding of DcPIV3 evolution and ecology, as well as its pathogenicity in camels.

## Data Availability Statement

The datasets presented in this study can be found in online repositories. The names of the repository/repositories and accession number(s) can be found at: https://www.ncbi.nlm.nih.gov/genbank/, MW545527; https://www.ncbi.nlm.nih.gov/genbank/, MW545528; https://www.ncbi.nlm.nih.gov/genbank/, MW504257; https://www.ncbi.nlm.nih.gov/genbank/, MW504258; https://www.ncbi.nlm.nih.gov/genbank/, MW504259; https://www.ncbi.nlm.nih.gov/genbank/, SRR13442189; https://www.ncbi.nlm.nih.gov/genbank/, SRR13442188; and https://www.ncbi.nlm.nih.gov/genbank/, SRR13442187.

## Author Contributions

JT, UW, and PW conceived and designed the experiment. JT, HL, JF, SJ, KL, SE, and K-HC performed the experiment. JT, UW, HL, JYHF, HC, SL, and PW contributed to analysis. JT, UW, HL, and PW drafted the manuscript. All authors reviewed and revised the first and final drafts of this manuscript. PW and UW are co-corresponding authors who contributed equally to this article.

## Funding

This work was partly supported by the Health and Medical Research Fund-Commissioned Research on Control of Infectious Diseases (Phase IV; CID-HKU1).

## Conflict of Interest

The authors declare that the research was conducted in the absence of any commercial or financial relationships that could be construed as a potential conflict of interest.

## Publisher’s Note

All claims expressed in this article are solely those of the authors and do not necessarily represent those of their affiliated organizations, or those of the publisher, the editors and the reviewers. Any product that may be evaluated in this article, or claim that may be made by its manufacturer, is not guaranteed or endorsed by the publisher.

## Footnotes

^1^ https://www.ncbi.nlm.nih.gov/orffinder/

## Supplementary Material

The Supplementary Material for this article can be found online at: https://www.frontiersin.org/articles/10.3389/fmicb.2021. 739779/full#supplementary-material
