# Supplementary material for: Co-circulation of a Novel Dromedary Camel Parainfluenza Virus 3 and Middle East Respiratory Syndrome Coronavirus in a Dromedary Herd With Respiratory Tract Infections
Source: Front Microbiol. 2021 Dec 7;12:739779. doi: 10.3389/fmicb.2021.739779 (PMC8705932; doi:10.3389/fmicb.2021.739779)
Supplement: Supplementary file 1 [file Table_1.DOCX]

**Supplementary Table S1**

Primers used for genome sequencing of DcMERS-CoV.

| Primer pairs | Primer name | Location | Primers (5’ – 3’) |
| --- | --- | --- | --- |
| 1 | LPW 26298  LPW 26124 | 1 -1112 | GATTTAAGTGAATAGCTTGGCTA  CCATCCTTTTGGACCACACTA |
| 2 | LPW 25991  LPW 25992 | 980 - 2,551 | CGTGCTGATGACGAAGGCTT  ATCTTGTTGAACGGACTTAGCCTT |
| 3 | LPW 25364  LPW 25365 | 2,480 - 3,517 | CAGGGAGTCTCTAATATTCCCAT  TCTTTTACCTCGGGCTGGAT |
| 4 | LPW 25993  LPW 25994 | 3,431- 4,725 | CCTGTTGTGCCTGATACTGTTGAA  AACTGCTGTGTCGTGCGTGAA |
| 5 | LPW 25995  LPW 25996 | 4,591 - 6,053 | GCTCCTGCATTGGTCTGATCAA  CCCAAAGAATTGGTTTGCCTTT |
| 6 | LPW 25366  LPW 25367 | 5,972 - 7,074 | CGATGTGTTGTTGGCTGAGTT  AATTACACATTGCAGAACGGTT |
| 7 | LPW 25997  LPW 25998 | 6,965 - 8,107 | TGTGACGGTCTTGCTTCAGCTT  TGCTGCGTCAATGAATGTTGTT |
| 8 | LPW 25999  LPW 26000 | 8,034 - 9,104 | TCTCTACTGCTCGTGATGGCGTA  TACCATCATACAAGTCGTAACGAA |
| 9 | LPW 25368  LPW 25369 | 9.019 - 10,014 | CATACTGCCATGATCCTACTGTT  ACACGCCAGAGGTTATGCTA |
| 10 | LPW 26001  LPW 26002 | 9,919 - 11,079 | AGCATGTCATCTTGCTAAAGCCTT  AGTGGGAACAACTGTGTGGGAA |
| 11 | LPW 26003  LPW 26004 | 10,984 - 12,061 | GACCCTTGTCTCAACCTATGTGAT  TTGAAGTACGCTAGGAGTGTCAAA |
| 12 | LPW 25370  LPW 25371 | 11,957 - 13,052 | TTTCGAGAAATTCGTAAGTCTCTT  AACGGTGAAGTTAACAAGTGACAA |
| 13 | LPW 26005  LPW 26006 | 12,961 - 14,559 | GCACATTGCTGCGACTGTTAGAT  GGCAATGTGCATGGCTGGAT |
| 14 | LPW 25529  LPW 25373 | 14,491 - 16,017 | CTCCATAGACATAGGCTCTCTCTT  AGAGGGTAAGCATCTATAGCCAA |
| 15 | LPW 21356  LPW 21357 | 15,051 - 15258 | GGTTGGGACTATCCTAAGTGTGA  ACCATCATCNGANARDATCATNA |
| 16 | LPW 26007  LPW 26008 | 15,931 - 17,010 | GGTTGCTTTGTAGATGATATCGTT  GTTGGCAACATGACTTGCGAA |
| 17 | LPW 26009  LPW 26010 | 16,946 - 18,088 | TTAAAATTACTGGGTTGTACCCAA  CAACACTAACGTATGTTGGTGCAT |
| 18 | LPW 25374  LPW 25375 | 17,990 - 19,035 | TACAAGCTCCAGTCTCAGATTGTA  ACAGGGTCATCAACAATAGGAA |
| 19 | LPW 26011  LPW 26012 | 18,956 - 20,518 | CTGCTCTTCTTGCCGGTTCAT  TGACAACCTTGGATACTACGCCAA |
| 20 | LPW 26013  LPW 26014 | 20,420 - 21,698 | CGGCTTTTAAGGCGGTGTGTT  CATGGTCTCCCTGATAGGGAAAAA |
| 21 | LPW 25376  LPW 25377 | 21,630 - 22,851 | CCTCAAGGCCGTACATATTCTA  GGGATATTGGACCAGCAGAA |
| 22 | LPW 26015  LPW 26016 | 22,728 - 24,117 | GTAGTCAAATATCTCCAGCAGCAA  CTACGTGCACTACGACTGCCAGTA |
| 23 | LPW 22624  LPW 25621 | 24,014 - 25,112 | GAAAAGCTCTCAATCATCTCC  CCTCCCTCCTCCTCTTCTCGGC |
| 24  25  26  27  28  29  30 | LPW 22628  LPW 22888  LPW 22887  LPW 25624  LPW 25622  LPW 25626  LPW 25625  LPW 25629  LPW 21391  LPW 21392  LPW 25630  LPW 21392  LPW 25630  LPW 418 | 24,998 - 26,326  26,186 - 27,063  26,557 - 27,577  27,469 - 28,691  28,565 - 29,797  29,157 - 29,797  29,157 - 30,000 | GGATTGTTGATGAGTGGTC  CCTTATGGCGGCCAACCT  CTGCGCTACGTTAAGCGTAG  CATAGTAAACTTTTTCATT  GCCTGTGTATAGAGTTAACAC  GTCCAAGAACGAATAGGGTTG  CGTGCCTGCAACGCGCGATTC  GTCCCTCTTACCTTTCCACC  CGAATCTCAATTTCATTGTTATG  GATCGCGGCAATCGTTTGTG  GGAAACTCTACCCGCGGCAC  GATCGCGGCAATCGTTTGTG  GGAAACTCTACCCGCGGCAC  CGGGAAGAGCTCTACAGTGT |
